# Overexpression of Nrf2 attenuates Carmustine-induced cytotoxicity in U87MG human glioma cells

**DOI:** 10.1186/s12885-015-1134-z

**Published:** 2015-03-13

**Authors:** Sangeetha Sukumari-Ramesh, Niyathi Prasad, Cargill H Alleyne, John R Vender, Krishnan M Dhandapani

**Affiliations:** 1 Department of Neurosurgery, Georgia Regents University, 1120 15th Street, CB2517, Augusta, GA 30912 USA; 2 1120 15th Street, CA1010, Augusta, GA 30912 USA

**Keywords:** Nrf2, Carmustine, BCNU, Glioma, Chemotherapy

## Abstract

**Background:**

Malignant glioma is one of the most devastating tumors in adults with poor patient prognosis. Notably, glioma often exhibits resistance to conventional chemotherapeutic approaches, complicating patient treatments. However, the molecular mediators involved in tumor chemoresistance remain poorly defined, creating a barrier to the successful management of glioma. In the present study, we hypothesized that the antioxidant transcription factor, Nrf2 (nuclear factor erythroid-derived 2 like 2), attenuates glioma cytotoxicity to Carmustine (BCNU), a widely used chemotherapeutic agent known to modulate cellular oxidative balance.

**Methods:**

To test the hypothesis, we employed human malignant glioma cell line, U87MG and overexpression of Nrf2 in glioma cells was achieved using both pharmacological and genetic approaches.

**Results:**

Notably, induction of Nrf2 was associated with increased expression of heme oxygenase-1 (HO-1), a stress inducible enzyme involved in anti-oxidant defense. In addition, over expression of Nrf2 in U87MG cells significantly attenuated the cytotoxicity of Carmustine as evidenced by both cellular viability assay and flow cytometry analysis. Consistent with this, antioxidants such as glutathione and N-acetyl cysteine significantly reduced Carmustine mediated glioma cytotoxicity.

**Conclusions:**

Taken together, these data strongly implicate an unexplored role of Nrf2 in glioma resistance to Carmustine and raise the possible use of Nrf2 inhibitors as adjunct to Carmustine for the treatment of malignant glioma.

**Electronic supplementary material:**

The online version of this article (doi:10.1186/s12885-015-1134-z) contains supplementary material, which is available to authorized users.

## Background

Malignant glioma is one of the most devastating tumors in adults. The worldwide annual incidence of malignant glioma is approximately 6 cases per 100,000 people [1] and each year, more than 14,000 new cases are being diagnosed in the United States. In contrast to other solid tumors, glioma presents various therapeutic challenges that include its intracranial location, aggressive biological behavior and infiltrative growth. Though multimodal treatment regiments are being used for the treatment of malignant glioma, it is often associated with poor patient prognosis and the mean life expectancy of patients is still less than 14 months [2].

Carmustine or bis-chloroethylnitrosourea (BCNU) wafer is the only FDA approved intracerebral chemotherapeutic agent for the treatment of newly diagnosed and recurrent malignant glioma [3]. After maximal surgical resection of tumors, biodegradable wafers of Carmustine (Gliadel®) are implanted inside the tumor cavity, providing an innovative way of delivering chemotherapy directly to the brain tumors with minimal systemic toxicity and greater efficacy than systemic Carmustine administration [4]. However, the recent studies demonstrated that the efficacy of Carmustine is substantially limited by chemoresistance [5]. Carmustine exerts tumor cytotoxicity via multiple mechanisms and it often interferes with DNA replication and transcription [6,7]. In addition, Carmustine is known to carbamylate lysine residues on proteins [8] causing protein carbamylation, a post translational protein modification that could irreversibly inactivate enzymes including glutathione reductase [9-11]. Therefore, by inhibiting glutathione reductase an enzyme that plays critical roles in cellular oxidative balance, Carmustine treatment may modulate the cellular oxidative status.

Nrf2 is a key redox-sensitive transcription factor that regulates the expression of endogenous antioxidants, phase II detoxification enzymes, and other cellular defensive proteins in response to cellular stress. The transcriptional activity of Nrf2 is negatively regulated by the cytoplasmic protein, Kelch-like ECH-associated protein 1 (Keap1) [12,13]. Under homeostatic conditions, Keap1 constitutively targets Nrf2 for ubiquitin conjugation and subsequent proteasome degradation in the cytoplasm by acting as a substrate adaptor for the Cul3-based E3 ubiquitin ligase complex [14]. Upon exposure of cells to oxidative stress or Nrf2 inducers such as tert-butylhydroquinone (TBHQ), multiple cysteine residues on Keap1 are alkylated, compromising the ability of Keap1 to efficiently ubiquitinate Nrf2 and resulting in elevated Nrf2 protein levels and transcriptional activity. Though recent studies demonstrated a role of Nrf2 in glioma invasion [15], angiogenesis [16,17], the self-renewal of glioma stem cells [18], and temozolomide-mediated cytotoxicity [19,20] its precise role in tumor progression remains largely controversial. Moreover, the functional role of antioxidant transcription factor Nrf2 in malignant glioma resistance to Carmustine remains largely uncharacterized. Altogether, given the role of Nrf2 in antioxidant defense mechanisms coupled with the potential modulation of cellular oxidative status by Carmustine treatment, we hypothesized that Nrf2 may functionally regulate tumor cell sensitivity to the cytotoxic effects of Carmustine, a widely used intracerebral chemotherapeutic agent.

## Methods

### Materials

All cell culture reagents, sera, and media were purchased from Hyclone Laboratories (Logan, UT). Carmustine (BCNU) and tert-butylhydroquinone (TBHQ) were purchased from Sigma-Aldrich Co (St. Louis, USA). TBHQ was dissolved in dimethyl sulfoxide (DMSO) and DMSO was used as a vehicle in all studies. MTT was purchased from Calbiochem (USA).

### Cell culture

Human U87MG malignant glioma cells (American Type Tissue Collection, Manassas, VA) were cultured in Dulbecco’s modified Eagle’s medium (DMEM) supplemented with 5% fetal bovine serum, 5% bovine growth serum, and antibiotics in a 37°C humidified incubator at 5% CO2.

### Cellular viability assay

MTT reduction assay was performed as an estimate of cellular viability, as described earlier [21]. Briefly, cells (3 × 10^4^ cells/well) were plated overnight in 24-well plates and treated with vehicle or TBHQ or Carmustine as detailed in respective figure legends. Following treatments, MTT (5 mg/mL; 50 μl/well) was added to each well and incubated for 4 h at 37°C. The wells were then emptied and the blue formazan salts were dissolved in acidic isopropanol (400 μl/well) and absorbance was measured using a plate reader (Biotek) at 540 nm using a reference wavelength of 690 nm. Cellular viability was normalized to vehicle treated control wells, which represented 100% viability.

### BrdU (bromodeoxyuridine) incorporation assay

Cell proliferation was measured by estimating BrdU incorporation using a Proliferation Assay kit (Calbiochem, Merk, Darmstadt, Germany), as per the manufacturer’s instructions. Briefly, U87MG cells were cultured overnight in 96-well plates at a density of 10^4^ cells/100 μl/well in complete growth media and treated with TBHQ/Vehicle for 24 h. BrdU label solution (Calbiochem) was added 4 h prior to the completion of TBHQ treatment. The anti-BrdU antibody was added and incubated for 1 h at room temperature and this was followed by 30 min incubation with the respective secondary antibody. The absorbance was read at 450 nm on a Synergy HT Biotech Elisa reader.

### Flow cytometry

Cell death was quantified by flow cytometry, as described previously by our group [22]. Briefly, the cells were plated overnight at density of 100,000 cells/well and treated with either vehicle or TBHQ (30 micromoles) for 6 h. The wells were then emptied and vehicle or Carmustine (50 μg/ml) was added and incubated for 18 h. Afterwards, the adherent and non-adherent cells were collected and washed and the cell suspensions were stained for 15 min at room temperature with annexin V-PE (BD Pharmigen, San Diego, CA), an early apoptotic marker, and with 7-aminoactinomycin D (7-AAD), a fluorescent marker that labels dead cells. The percentage of apoptotic or necrotic cell death was quantified using a FACScan flow cytometry.

### Immunocytochemistry

U87MG cells after treatment with either vehicle/TBHQ (30 μM) for 6 h were fixed with ice cold methanol for 5 minutes. Cellular fixation was followed by washing twice with PBS and a 10 min treatment with 0.1% Triton‐X 100 in PBS. Cells were then incubated with 12% donkey serum for 1 h at room temperature to block any nonspecific binding of antibodies. Primary antibody [Nrf2 (1:100; Santa Cruz Biotechnology, Santa Cruz, CA)] incubation was carried out for 18 h at 4°C, and this was followed by secondary antibody (Alexa Fluor) incubation for 2 h at room temperature. Finally, cells were cover slipped with a mounting medium containing nuclear stain DAPI and immunofluorescent analysis was performed using a LSM510 Meta confocal laser microscope (Carl Zeiss, Thornwood, NY, USA).

### Western blotting

Western blotting was performed as described by our laboratory [21,22]. Briefly, cells after respective treatment were washed with phosphate buffered saline (PBS) and whole cell lysates were collected in radioimmunoprecipitation (RIPA) buffer containing protease inhibitor cocktail, and phenyl methane sulfonyl fluoride (PMSF). Cell lysates were sonicated, centrifuged for 5 min at 14,000 rpm at 4°C, and protein concentrations were quantified by BCA protein assay kit (Pierce, Rockford, IL). Thirty micrograms of protein was resolved on a 4–20% sodium dodecyl sulfate–polyacrylamide gel and transferred onto a polyvinylidene difluoride (PVDF) membrane. Blots were incubated overnight at 4°C in respective primary antibody [Nrf2 (1: 250) Santa Cruz Biotechnology, Santa Cruz, CA), heme oxygenase-1 (1: 1000; Abcam, Cambridge, MA), or β-actin (1:3000; Sigma, St Louis, MO)] followed by a 2-h incubation with a corresponding Alexa Fluor secondary antibody. Blots were visualized using the Li-Cor Odyssey near-infrared imaging system and quantified using Quantity One software (Bio-Rad, Foster City, CA).

### Over expression of Nrf2

Precision LentiORF lentiviral particles (Thermo scientific Open Biosystems) were used to overexpress Nrf2 in U87MG cells as per manufacturer’s recommended protocol. Briefly, U87MG cells were transduced with lentiviral particles at MOI (Multiplicity of infection) of 1.8. Media was replaced 72 h later with growth media and after 48 h, cells were challenged with blasticidin S (5 μg/mL; the minimum concentration required to kill non-transduced U87MG cells). Blasticidin S selection continued for one week, with media replenishment thrice weekly. The Blasticidin S resistant cells were collected and Western blotting was performed to ensure stable Nrf2 overexpression. Control cells were stably transduced with lentiviral particles containing Red Fluorescent Protein (RFP).

### ELISA-based measurement of Nrf2 activity

The TransAM Nrf2 Kit (Active Motif; California, USA) was used to assay the DNA-binding activity of Nrf2 in the nuclear extracts of both the RFP and Nrf2-overexpressed cells. In brief, 5 μg of nuclear extract prepared using nuclear extraction kit (Active Motif, USA) was incubated in a 96-well plate that was coated with oligonucleotide containing a consensus binding site for Nrf2. After 1 h of incubation, the wells were incubated with 100 μl of a 1:1000 dilution of Nrf2 antibody. This was followed by incubation with 100 μl of a 1:1000 dilution of horseradish peroxidase-conjugated secondary antibody at room temperature. The wells were developed using 100 μl of developing solution for 10 min before the addition of 100 μl of stop solution. Optical density was read at 450 nm with a reference wavelength of 650 nm using a Synergy HT Biotech Elisa reader.

### Statistical analysis

For cellular viability studies, n = 4 wells/group were used within each experiment for analysis. For western blotting, all experiments were performed at least in triplicate using independent cell cultures. All experiments were repeated at least three times for the validation of results. Data was analyzed using a one-way analysis of variance (ANOVA), followed by Student–Newman–Keul’s or Dunnett’s post-hoc test. A P value, p < 0.05 was considered to be statistically significant.

## Results

### TBHQ up regulated transcription factor Nrf2 in U87MG glioma cells

**Figure 1:**
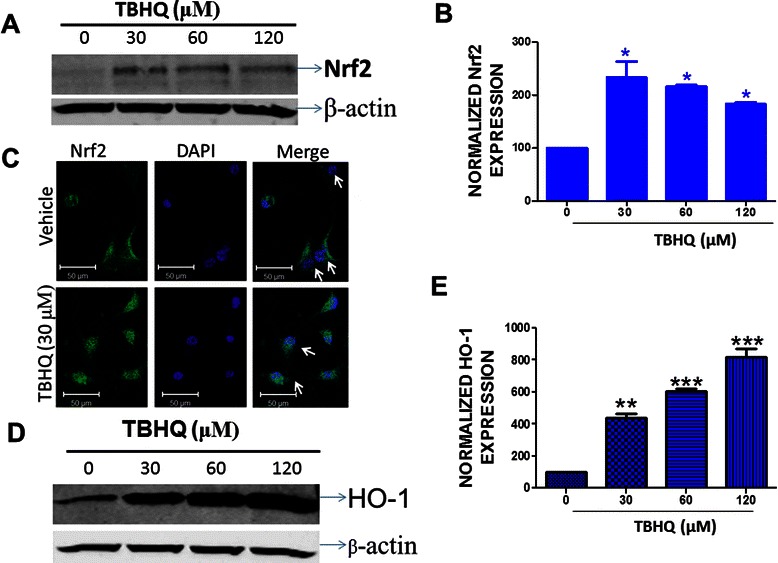
**TBHQ and Nrf2 upregulation.** U87MG cells were treated with either vehicle or TBHQ for 6 h and the induction of Nrf2 (MW: ~102 kDa) was quantified using **(A)** western blotting followed by **(B)** densitometry analysis. The immunocytochemistry analysis followed by **(C)** confocal imaging further confirmed induction and nuclear translocation of Nrf2 in glioma cells by TBHQ (scale bar = 50 μm). TBHQ treatment of glioma cells also resulted in the induction of HO-1 (MW : ~31 kDa), one of the Nrf2 regulated molecular targets, as evidenced by **(D)** western blotting followed by **(E)** densitometry analysis. Densitometry is expressed as the mean ± SEM from three independent trials and data were analyzed using One-way ANOVA followed by Dunnett’s post-hoc test (* p < 0.05, ** p < 0.01, *** p < 0.001 vs. vehicle-treated cultures).

**Figure 2:**
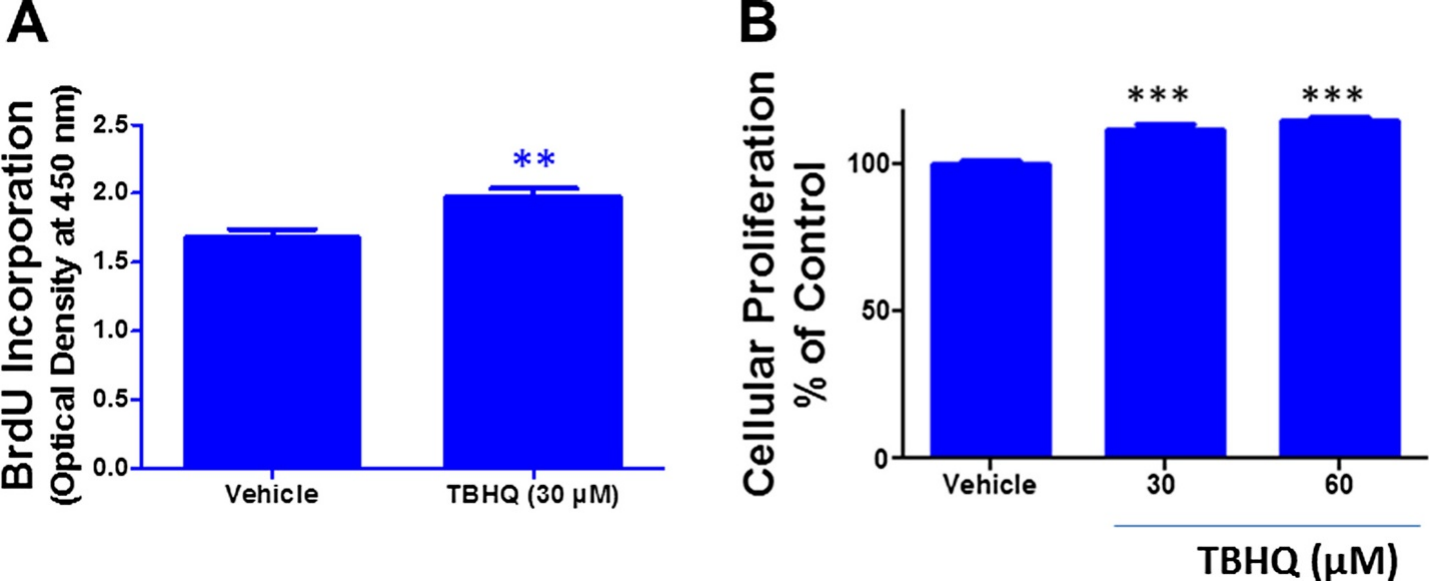
**TBHQ and glioma cell proliferation.** U87MG cells were treated with either vehicle or TBHQ for 24 h and the cellular proliferation was assessed using **(A)** BrdU incorporation assay and **(B)** MTT assay as described in Methods. Data are representative of at least three independent trials (n = 3/trial) and are expressed as mean ± SEM. ** p < 0.01, *** p < 0.001 vs. vehicle-treated cultures.

To establish the role of transcription factor Nrf2 in glioma cells, we employed an Nrf2 inducer, TBHQ. We found that TBHQ treatment (30-120 μM) significantly augmented the protein expression of Nrf2 in U87MG cells (Figure 1A and B). A 6 h treatment with 30 μM of TBHQ resulted in 133% ± 30 (p < 0.05 vs. vehicle) increase in Nrf2 levels in U87MG cells in comparison to vehicle treated cells (Figure 1B). Immunocytochemical analysis reaffirmed the induction of Nrf2 upon TBHQ treatment. Notably, the TBHQ treatment also resulted in enhanced nuclear translocation of Nrf2 as evidenced by increased colocalization of Nrf2 with the nuclear stain, DAPI in comparison to control (Figure 1C). The expression of Heme oxygenase 1 (HO-1), one of the potential downstream targets of Nrf2, was next analyzed. The results showed that U87MG cells constitutively express the HO-1 protein (Figure 1D). Moreover, U87MG cells treated with TBHQ exhibited a significant increase in HO-1 expression (Figure 1D and E), suggesting TBHQ mediated upregulation of Nrf2 transcriptional activity. Given the role of redox mechanisms in tumor cell proliferation coupled with the role of Nrf2 in cellular antioxidant defense mechanisms [23], we first questioned whether Nrf2 induction in glioma cells modulates cellular proliferation. To this end, the effect of TBHQ on the proliferation of U87MG cells was evaluated by BrdU incorporation assay. TBHQ treatment augmented BrdU incorporation in glioma cells by 17.5% (p < 0.01 vs. vehicle) as compared to vehicle treatment (Figure 2A). Moreover, the MTT proliferation assay further validated the increase in glioma cell proliferation by TBHQ (Figure 2B) and demonstrated 11.5 and 14.7% increase in proliferation upon 30 and 60 μM of TBHQ treatment, respectively.

### TBHQ attenuated Carmustine induced cytotoxicity in glioma cells

**Figure 3:**
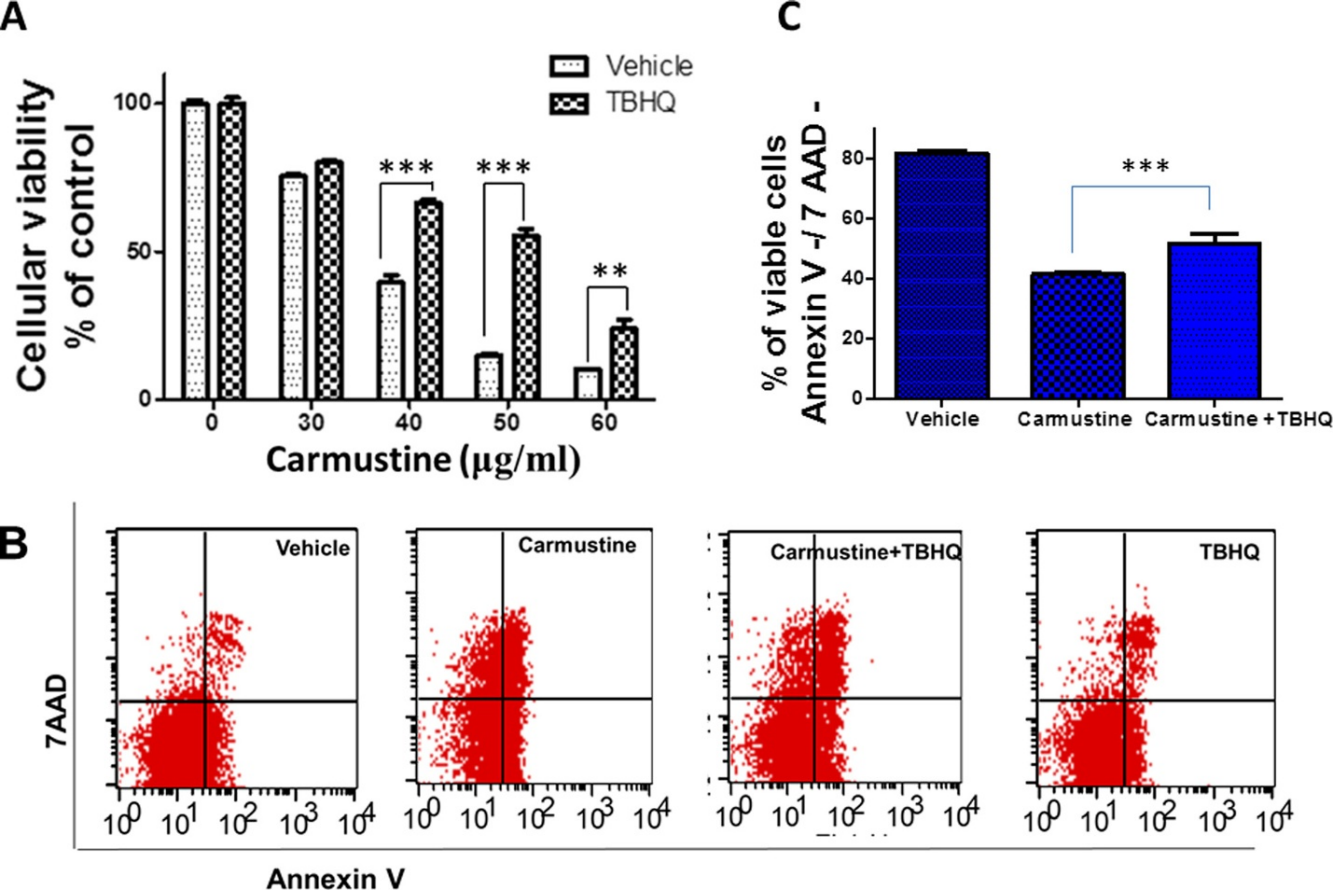
**TBHQ and resistance to Carmustine.** U87MG cells were treated with either vehicle or TBHQ (30 μM) for 6 h. After respective treatment, the media were removed, cells were replenished with media containing either vehicle or Carmustine at indicated concentrations and incubated for 18 h. The cell viability was measured using **(A)** MTT reduction assay and **(B)** flow cytometry analysis. Data from MTT Assay are representative of three independent experiments and are expressed as mean ± SEM. ** p < 0.01, *** p < 0.001 vs. vehicle treated cells. For flow cytometry analysis, the cells after treatments were collected and stained with 7-AAD (y-axis) a marker of cell death and Annexin V (x –axis), a marker of early apoptotic cell death. The percentages of viable cells **(C)** which are both Annexin V/7-AAD negative are shown. Data are representative of two independent experiments and are expressed as mean ± SEM. *** p < 0.001 vs. Carmustine alone treated cells.

To delineate the role of Nrf2 in chemoresistance to Carmustine, we pre-treated U87MG cells with TBHQ for 6 h and the cytotoxic response to Carmustine was studied. TBHQ pre-treated U87MG cells exhibited significantly lower cytotoxicity to Carmustine in comparison to vehicle treated cells (Figure 3A). Along these lines, Carmustine (50 μg/ ml) induced 85.26 ± 0.8761% cytotoxicity in U87MG cells and upon TBHQ pre-treatment the Carmustine mediated cytotoxicity was reduced to 44.64 ± 2.325% (p < 0.001 vs. Carmustine treatment alone) (Figure 3A). Moreover, the 6 h pre-treatment with TBHQ did not significantly increase the proliferation of U87MG cells in comparison to vehicle treated controls (n = 8; data not shown) suggesting the role of Nrf2 mediated antioxidant signaling independent of proliferation in attenuating Carmustine mediated cytotoxicity. Furthermore, similar results were obtained in another human malignant glioma cell line, U118 (Additional file 1: Figure S1), reaffirming the role of Nrf2 in Carmustine mediated cytotoxic effects in glioma. The cellular viability studies were further verified using flow cytometry analysis (Figure 3B), which demonstrated a significant reduction in Carmustine mediated cytotoxicity upon TBHQ treatment (Figure 3C).

### NRF2 over expression in glioma cells induced resistance to Carmustine mediated cytotoxicity

**Figure 4:**
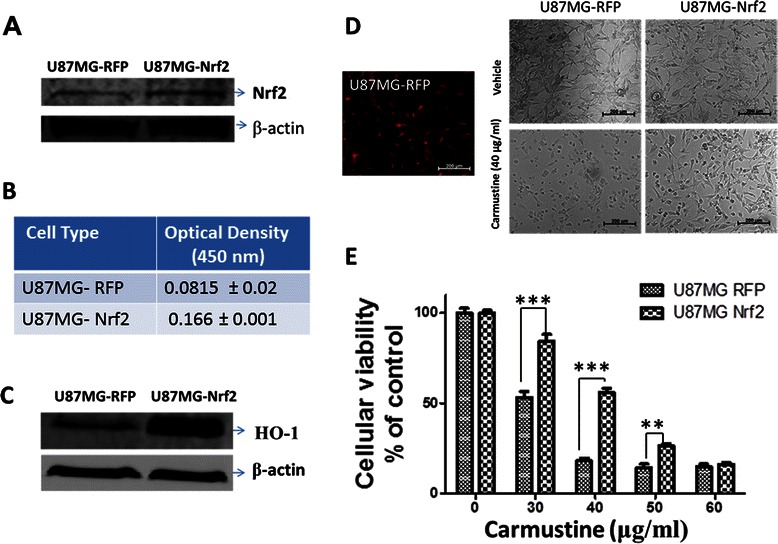
**Nrf2 and resistance to Carmustine.** U87MG cells were stably transduced with the Precision LentiORF lentiviral particles to accomplish Nrf2 (MW: ~102 kDa) overexpression evidenced by **(A)** western blotting. The genetic over expression of Nrf2 resulted in increased Nrf2 transcriptional activity as assessed by **(B)** TransAM Elisa and augmented expression of HO-1 as assessed by western blotting **(C)**. The confocal image **(D)** illustrates U87MG cells overexpressed with Red Fluoresent protein (RFP) that served as experimental control (scale bar = 200 μm). The U87MG cells stably over expressing either RFP or Nrf2 were subjected to ether vehicle or Carmustine treatment for 18 h and the cellular viability was assessed by **(E)** MTT reduction assay and the Figure 4D demonstrates the cellular morphology of cells upon Carmustine treatment using bright field microscopy (Scale bar =200 μm). Data are representative of at least three independent trials (n = 3/trial) and are expressed as mean ± SEM. ** p < 0.01, *** p < 0.001 vs. vehicle-treated cultures.

**Figure 5:**
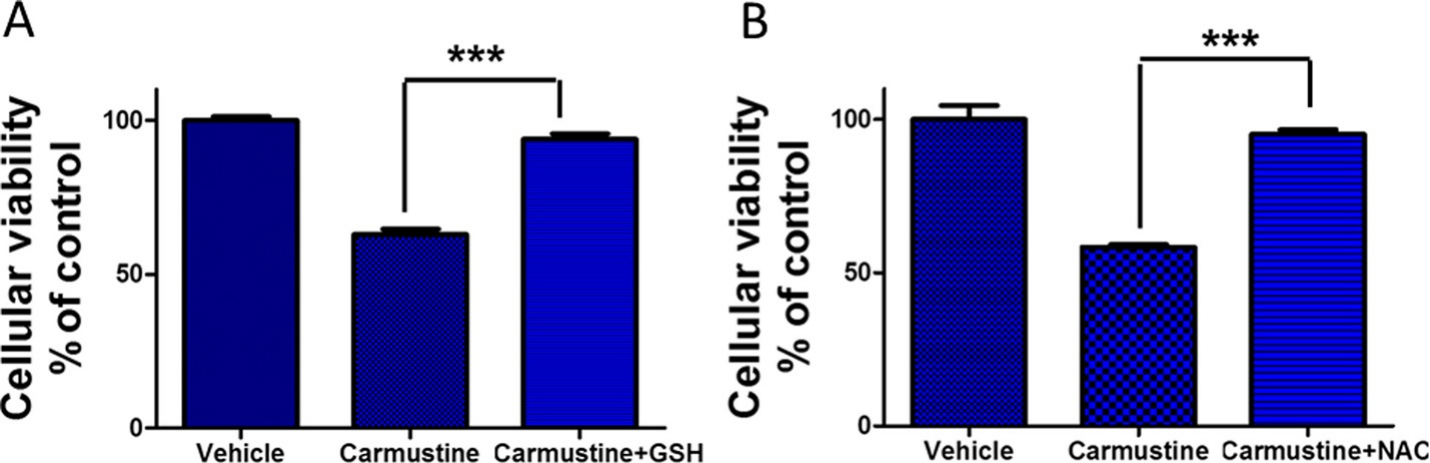
**Antioxidants and Carmustine cytotoxicity.** U87MG cells were treated with Carmustine (40 μg/ml) for 18 h in the presence of either **(A)** glutathione (GSH;5 mM) or **(B)** N-acetyl cysteine (NAC; 5 mM) and cellular viability was quantified using MTT assay. Data are representative of at least three independent trials (n = 3/trial) and are expressed as mean ± SEM. *** p < 0.001 vs. Carmustine alone treated cultures.

To further establish the role of Nrf2 in Carmustine resistance, we performed genetic overexpression of Nrf2 in U87MG cells using lentiviral particles. The induction of Nrf2 by lenti viral particles was confirmed by western blotting analysis (Figure 4A). Cells transduced with lentiviral particles containing Red Fluorescent Protein (RFP) served as the experimental control (Figure 4D; left panel). In addition, a TransAM ELISA was performed to validate the DNA binding activity of Nrf2 upon genetic overexpression. As shown in Figure 4B, a 103.7% increase in DNA binding activity of Nrf2 was found in the nuclear extracts derived from Nrf2 overexpressed cells in comparison to RFP overexpressed cells. In addition, genetic over expression of Nrf2 was associated with the induction HO-1 further confirming Nrf2 mediated regulation of HO-1 in U87MG glioma cells (Figure 4C). More importantly, Nrf2 overexpressed cells exhibited significantly lower cytotoxicity to Carmustine in comparison to RFP over expressed cells (Figure 4D and E). To further explore the role of antioxidant mechanisms in tumor cell resistance to Carmustine, we studied the role of antioxidants such as glutathione and N-acetyl cysteine in Carmustine mediated cytotoxic effects. Interestingly, we found that both glutathione and N-acetyl cysteine significantly attenuated Carmustine mediated cytotoxicity in U87MG cells. Carmustine treatment alone induced 39.4% cytotoxicity in U87MG cells, whereas co treatment of Carmustine with glutathione or N-acetyl cysteine the induction of cytotoxicity was reduced to 6.01 and 4.87% respectively (Figure 5A and B).

## Discussion

Though surgical resection is one of the prime treatment options for malignant glioma, the complete surgical removal of the tumor is often a challenge owing to the infiltrative nature of glioma. Thus, the treatment strategy frequently demands chemotherapeutic approaches for improved patient outcomes and a better understanding of the underlying mechanisms of chemoresistance is therefore critical. The intracerebral delivery of Carmustine using Carmustine wafers is found to be very well tolerated in patients and it allows drug release in a constant manner with minimal systemic toxicity [24]. However, Carmustine failed to substantially prolong median survival of GBM patients. The reason for this unsatisfactory clinical outcome remains unclear but may involve intrinsic/acquired chemoresistance of the tumor cells. To this end, variations in multidrug resistance genes [25-27] and DNA repair activity [28] have been demonstrated to play roles in glioma resistance to Carmustine. Although, several studies have shown that a deficiency of DNA repair enzyme, O6-methylguanine methyl-DNA transferase (MGMT) can increase the sensitivity of glioma to Carmustine [29-31], many tumors with low levels of MGMT are nevertheless chemoresistant [32,33], suggesting the involvement of other unknown mechanisms of chemoresistance.

Though Nrf2 has been implicated in chemoresistance to 5-fluorouracil, carboplatin, cisplatin and temozolomide [20,34-37], the role of Nrf2 in glioma resistance to Carmustine remained largely uncharacterized. Herein, we report for the first time that both pharmacological and genetic upregulation of Nrf2 in U87MG grade IV malignant glioma cells significantly attenuate Carmustine mediated cytotoxicity. This finding has significant clinical implications, given that tumor cells often exhibit elevated metabolic rate and may have augmented Nrf2 level as a result of tumor cell adaptation to high metabolic demand. Along these lines, a number of malignant tumors such as lung, ovarian, colon, breast and pancreatic cancer, exhibit an increased transcriptional activity of Nrf2 [38-43]. Our findings also demonstrate that as a consequence of enhanced Nrf2 expression, glioma cells could acquire augmented cellular proliferation. In addition, a recent study also identified a role of Nrf2 in promoting tumor angiogenesis through the HIF-1 α/ VEGF pathways [16]. Altogether, strategies to pharmacologically attenuate the Nrf2 levels and/or activity may reduce glioma growth and resistance to Carmustine.

Keap1, a BTB-Kelch protein, is regarded as the principal and negative regulator of Nrf2 and several protein kinase pathways, including mitogen-activated protein kinase and protein kinase C, have been implicated in transducing signals that control Nrf2 dependent gene expression [44,45]. Promoter methylation of KEAP1 gene is found in malignant glioma [46] and a strong inverse correlation is discovered between methylation levels and KEAP1 mRNA transcript in tumor tissue [46] suggesting a reduced expression of KEAP1 in glioma. In line with this, both U87MG and T98G glioma cells (data not shown) express basal protein expression of Nrf2. Nrf2 is believed to exert its transcriptional function by forming heterodimers with small Maf (v-maf musculoaponeurotic fibrosarcoma oncogene family) proteins, and binding to ARE-containing gene promoters [14]. Our studies demonstrate that Nrf2 regulates the expression of HO-1 in glioma cells. HO-1 is a rate-limiting enzyme that catalyzes heme degradation in which oxidative cleavage of the porphyrin ring results in the generation of biliverdin (antioxidant), carbon monoxide (anti apoptotic), and free iron [47,48]. HO-1 belongs to heat shock protein family and its expression is triggered by various cellular stress stimuli such as reactive oxygen species, hypoxia and heavy metals [49,50]. Owing to the antioxidant and cytoprotective nature of the enzymatic products of HO-1, elevated HO-1 expression due to deregulated Nrf2 signaling could protect tumor cells from oxidative stress-related injury and function as a key component of tumor cell adaptation to oxidative stress induced by chemotherapeutic agents [51]. Therefore, further characterization of Nrf2-HO-1 signaling is highly warranted and may lead to the development of novel therapeutic strategies for malignant glioma.

Apart from heme oxygenase 1 (HO-1), the best-characterized Nrf2 downstream genes include glutathione biosynthesizing enzymes such as glutathione S-transferases A1 and glutamate-cysteine ligase. For instance, Nrf2 knockout mice exhibit reduced expression of both detoxification enzymes and antioxidants [52,53]. Oxidative stress is widely implicated in the etiology of cancer and results from an imbalance in the production of Reactive Oxygen Species (ROS) and cell’s own antioxidant defenses. ROS are found elevated during cancer and have been shown to activate signaling pathways involved in cellular proliferation and migration [23]. Carmustine-mediated malignant glioma cell death was significantly attenuated by co-treatment with antioxidants such as N-acetyl-L-cysteine and glutathione, suggesting the prominent role of oxidative stress mechanisms in conferring Carmustine-mediated cytotoxicity. Many chemotherapeutic agents produce cytotoxic effects via generation of ROS and/or electrophilic actions, which lead to oxidative stress [54-56]. To this end, Carmustine is known to inhibit glutathione reductase [57,58], an integral component of the antioxidant defense mechanisms. Therefore, antitumor agents may also activate Nrf2 antioxidant signaling in tumor cells in a ROS-dependent manner, leading to the development of acquired chemoresistance. Though antioxidant vitamins such as retinoids, vitamin C, vitamin E and carotenoids have been extensively investigated in cancer prevention, the role of these vitamins in attenuating the efficacy of chemotherapeutic agents needs to be elucidated. In addition, Nrf2 is also known to regulate the expression of cysteine/glutamate exchange transporter, xCT that maintains extracellular glutamate levels [59]. Therefore, future studies are warranted to demonstrate the role of Nrf2 in regulating xCT expression and/or activity in glioma, as augmented levels of glutamate may facilitate tumor growth by eliciting neuronal damage. Altogether, Nrf2 may represent a very effective and potent therapeutic target for glioma and pharmacological inhibitors of Nrf2 may serve as a useful adjunct to Carmustine for the treatment of malignant glioma.

## Conclusion

In conclusion, we have demonstrated a novel role of Nrf2 in malignant glioma cell resistance to Carmustine. Altogether, the data suggest that antioxidant transcription factor Nrf2 might be a potent and viable molecular target for the treatment of malignant glioma.

## Additional file

Additional file 1: Figure S1. Human U118 malignant glioma cells (kindly donated by Dr. Raghavan Raju, Allied Health Sciences, Georgia Regents University) were cultured (3 × 10^4^ cells/well in 24 well plate) in Dulbecco’s modified Eagle’s medium (DMEM) supplemented with 5% fetal bovine serum, 5% bovine growth serum, and antibiotics in a 37°C humidified incubator at 5% CO2 and were treated with either vehicle or TBHQ (30 μM) for 6 h. After respective treatment, the media were removed; cells were replenished with media containing either vehicle or Carmustine at indicated concentrations and incubated for 18 h. The cell viability was measured using MTT reduction assay. Data from MTT Assay are representative of three independent experiments and are expressed as mean ± SEM. *** p < 0.001 vs. vehicle treated cells.
