# Supplementary figures and images for: Overexpression of Nrf2 attenuates Carmustine-induced cytotoxicity in U87MG human glioma cells
Source: BMC Cancer. 2015 Mar 13;15:118. doi: 10.1186/s12885-015-1134-z (PMC4365816; doi:10.1186/s12885-015-1134-z)

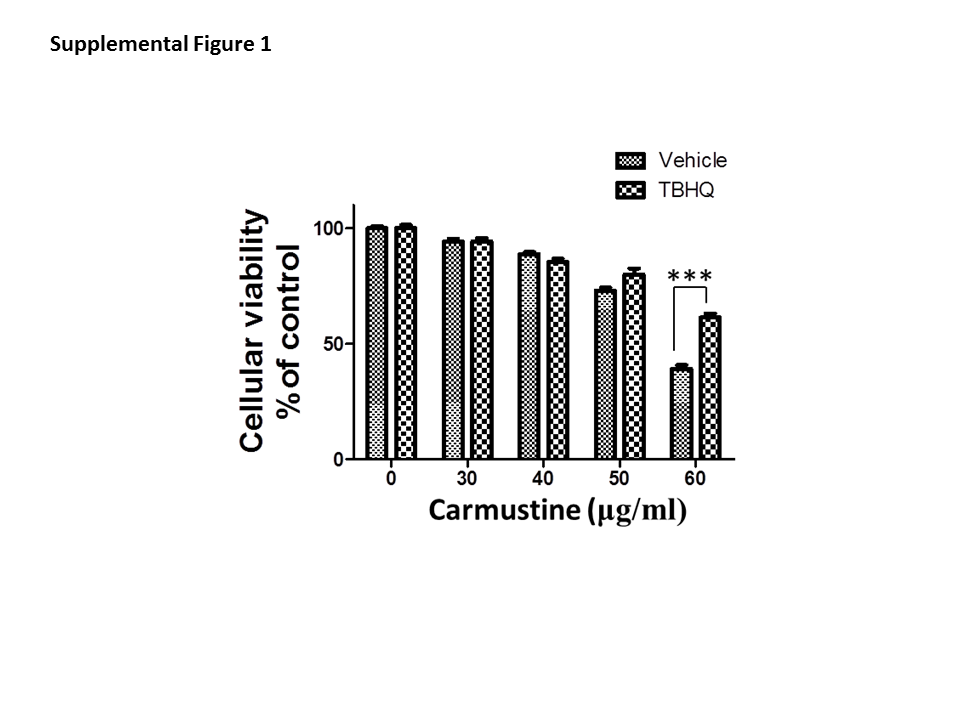

Supplement: Additional file 1: Figure S1. — Human U118 malignant glioma cells (kindly donated by Dr. Raghavan Raju, Allied Health Sciences, Georgia Regents University) were cultured (3 × 10^4^ cells/well in 24 well plate) in Dulbecco’s modified Eagle’s medium (DMEM) supplemented with 5% fetal bovine serum, 5% bovine growth serum, and antibiotics in a 37°C humidified incubator at 5% CO2 and were treated with either vehicle or TBHQ (30 μM) for 6 h. After respective treatment, the media were removed; cells were replenished with media containing either vehicle or Carmustine at indicated concentrations and incubated for 18 h. The cell viability was measured using MTT reduction assay. Data from MTT Assay are representative of three independent experiments and are expressed as mean ± SEM. *** p < 0.001 vs. vehicle treated cells. [file 12885_2015_1134_MOESM1_ESM.tiff]
